# Refining Metabolic Mass Transfer for Efficient Biosynthesis of Plant Natural Products in Yeast

**DOI:** 10.3389/fbioe.2021.633741

**Published:** 2021-03-05

**Authors:** Haijie Xue, Wentao Sun, Ying Wang, Chun Li

**Affiliations:** ^1^Institute of Biochemical Engineering, School of Chemistry and Chemical Engineering, Beijing Institute of Technology, Beijing, China; ^2^Key Lab for Industrial Biocatalysis, Ministry of Education, Department of Chemical Engineering, Tsinghua University, Beijing, China

**Keywords:** plant natural products, intracellular mass transfer, trans-plasma membrane mass transfer, metabolic engineering, yeast

## Abstract

Plant natural products are important secondary metabolites with several special properties and pharmacological activities, which are widely used in pharmaceutical, food, perfume, cosmetic, and other fields. However, the production of these compounds mainly relies on phytoextraction from natural plants. Because of the low contents in plants, phytoextraction has disadvantages of low production efficiency and severe environmental and ecological problems, restricting its wide applications. Therefore, microbial cell factory, especially yeast cell factory, has become an alternative technology platform for heterologous synthesis of plant natural products. Many approaches and strategies have been developed to construct and engineer the yeast cells for efficient production of plant natural products. Meanwhile, metabolic mass transfer has been proven an important factor to improve the heterologous production. Mass transfer across plasma membrane (trans-plasma membrane mass transfer) and mass transfer within the cell (intracellular mass transfer) are two major forms of metabolic mass transfer in yeast, which can be modified and optimized to improve the production efficiency, reduce the consumption of intermediate, and eliminate the feedback inhibition. This review summarized different strategies of refining metabolic mass transfer process to enhance the production efficiency of yeast cell factory (Figure 1), providing approaches for further study on the synthesis of plant natural products in microbial cell factory.

## Introduction

Plant natural products are secondary metabolites with complex molecular structures and inherent bioactivities (Williams et al., 1989), including a variety of organic compounds, such as flavonoids, terpenes, saponins, alkaloids, and sterols, which are widely used in medicine, food, perfume, and cosmetic (Mizutani et al., 1994; Enserink, 2005; Peraltayahya et al., 2011; Xu et al., 2017). Although these plant natural compounds and their derivatives display a broad range of applications in many areas, the current production mode mainly relies on extraction from plants, which is costly and complex because of the low concentration and plentiful structural analogs in their native producers (Nour et al., 2009). The low concentration and complex purification process limit the scale-up production of plant natural compounds via phytoextraction or chemical synthesis, failing to meet the market demand.

Fortunately, synthetic biology and system biology provide alternative approaches to solve such problems, which has facilitated the heterologous synthesis of plant natural products by microbial cell factory in the passing two decades (Shan et al., 2005; Moses et al., 2014; King et al., 2016). Compared with traditional extraction and chemical synthesis, the usage of fast-growing microorganisms dramatically shortens the production cycle, reduces environmental pollution, and simplifies the separation process for the synthesis of single structural compound in microbial cell factory (Sun et al., 2020). Many metabolic engineering strategies including overexpression of key enzymes, knocking out of competition pathway, and enzyme engineering have been employed to produce different kinds of plant natural products, such as amorphadiene (Reddingjohanson et al., 2011), farnesene (Su et al., 2015; Meadows et al., 2016; Yang et al., 2016), bisabolene (Kirby et al., 2014), miltiradiene (Hu et al., 2020), naringin (Park et al., 2009; Lv et al., 2019), kaempferol (Trantas et al., 2009), and coumarin (Ro and Douglas, 2004). Among these strategies, efforts to refining metabolic mass transfer of microorganisms (Noorman, 2001) by forming substrate channel or metabolic compartmentalization have been proven to play important roles on improving the production of plant natural products. For instance, the heterologous synthesis of tropane alkaloids was enhanced by refining metabolic mass transfer (Srinivasan and Smolke, 2020). Nowadays, many microorganisms have been used as chassis to produce plant natural products. Yeast, especially *Saccharomyces cerevisiae*, is one of the most favorite hosts for its inherent characteristics such as abundant precursors and complete endomembrane system (Li et al., 2018). As a GRAS (generally recognized as safe) strain with good robustness, yeast has been widely used in food and pharmaceutical production (Paddon et al., 2013; Khan et al., 2015). According to the space where transfer happens, the metabolism mass transfer in yeast can be divided into two types, which are the intracellular mass transfer (Lodish, 1988) and the trans-plasma membrane mass transfer (Whittam and Wheeler, 1970). In this article, recent advances about metabolic engineering strategies of plant natural compounds, especially optimization of metabolic mass transfer in yeast cell factory, were reviewed. Insights of engineering yeast as an efficient platform for product synthesis are also provided. Moreover, we illustrated the great potential for the synthesis of plant natural compound in yeast.

## Intracellular Mass Transfer in Yeast Cell Factory

**FIGURE 1:**
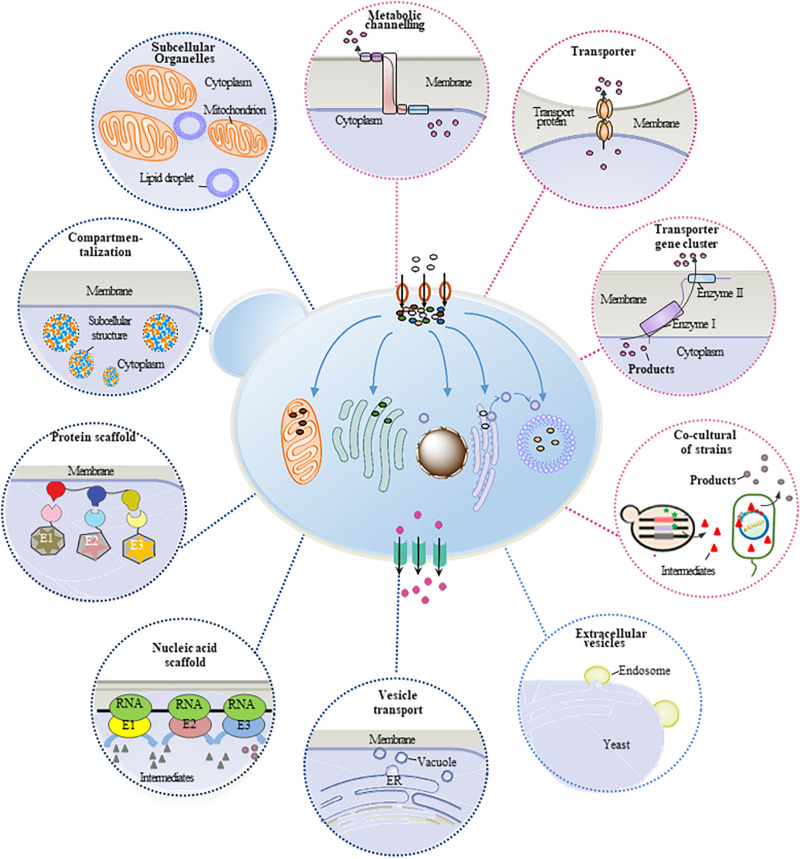
Transfer process of engineered *S. cerevisiae* in the synthesis of plant natural products. The brown circle with black arrow indicates substrate transferred into the cells. The green square with black arrow indicates the product transferred out of the cell. The blue arrow indicates intermediates transferred in cell. The circles around the cell represent different strategies to refine the metabolic mass transfer in *S. cerevisiae*. Blue circles represent strategies to enhance intracellular mass transfer process. Pink circles represent strategies to enhance trans-plasma membrane mass transfer process.

Intracellular metabolic mass transfer is an essential part of cell metabolism, which mainly refers to the substance’s delivery between enzymes and organelles. It is also one of the key factors to develop efficient microbial cell factory containing biosynthetic pathways with multienzyme reactions. The regulation of metabolic flux of the biosynthetic pathway and the spaces of different enzymes have been proven efficient ways for the refining of intracellular mass transfer. The production of plant natural products can be thus improved (Figure 1). In recent years, to boost the production of plant natural products in yeast cell factory, several strategies including protein fusion (Zhou et al., 2012) and artificial scaffolds (Wang and Yu, 2012) have been developed to strengthen the efficiency of mass transfer especially improve the catalytic efficiency of multi-enzyme reactions. In some cases, plant natural products and their intermediates are toxic to yeast cells (Valachovic et al., 2016), and some intermediates have feedback inhibitions to enzymes. To solve these issues, regulation strategies such as enzyme compartmentalization (Avalos et al., 2013) and suborganelles (Smith et al., 2000) were used to restrict intermediate distribution and redirect them toward the catalytic enzymes. As a result, the cytotoxicity was reduced, and the production was promoted. Here, several strategies such as optimizing the space location of enzymes and substances and enhancing the intracellular mass transfer in yeast cell factory have been summarized.

### Fusion Expression of Enzymes Shortens the Distance of Metabolite Diffusion

The biosynthetic pathway of plant natural compounds usually contains multienzyme reactions and locates in different organelles, which leads to some challenges in mass transfer efficiency and the toxicity of intermediates in microorganisms. Fusion expression is an effective way to shorten the distance between two proteins. Linking different genes together to obtain fusion protein is the most common strategy to colocalize enzymes of long pathway. In this way, the spatial location of enzymes can be adjusted to reduce cytotoxicity of intermediates and then to increase the intermediates transfer efficiency between different enzymes. For example, the 4-coumarate CoA-ligase (4CL), stilbene synthase (STS), and tyrosine ammonia lyase (TAL) were necessary enzymes in the polyketide resveratrol biosynthetic pathway in yeast. When the 4CL and STS were expressed in a fusion protein, the yield of resveratrol was increased by 15-fold (Zhang et al., 2006). The titer of patchoulol was increased by two times when the fusion expression of patchoulol synthase and FPP synthase was employed in *S. cerevisiae* (Albertsen et al., 2011). By fusion expression of SmCPS and SmKSL, as well as fusing the GGPP synthase and farnesyl diphosphate synthase, the metabolic flux of miltiradiene synthetic pathway was enhanced, and the titer of miltiradiene reached 488 mg/L in a 15-L bioreactor (Zhou et al., 2012). To improve geraniol production, the synthesis of geranyl diphosphate (GPP), the precursor of geraniol, was regulated by controlling the expression of endogenous ERG20, coupled with up-regulation of the mevalonate pathway by co-overexpressing IDI1, tHMG1, and UPC2-1 (Zhao et al., 2016). Moreover, the fusion expression of the key enzymes was improved by optimizing the amino acid linker and the order of the proteins, resulting in a production of 293 mg/L geraniol in fed-batch cultivation (Zhao et al., 2016). The fusion expression of enzymes can adjust the intracellular metabolic mass transfer of synthetic pathways, which suggests that adjusting physical localization of key enzymes can shorten the distance of metabolite diffusion and thus facilitate the yield of production.

### Utilization of Artificial Scaffolds Forms the Substrate Channeling

The utilization of fusion protein is a relatively simple and efficient way to regulate intracellular mass transfer. However, there are still some problems. For instance, in some cases, the fusion protein will form inclusion body without enzyme activities. More frequently, fusion expression may reduce the enzyme activity and destroy the structure of enzymes (Wheeldon et al., 2016; Poshyvailo et al., 2017). To solve these problems, some artificial scaffolds (including nucleic acid scaffolds and protein scaffolds) have been developed to form substrate channeling for the cascade enzymatic reactions, which can enhance the membrane free mass transfer process and improve the mass transfer efficiency. Han employed the retrotransposon element Ty1 as a scaffold to spatially organize enzymes involved in biosynthesis of farnesene and farnesol, forming the substrate channel and resulting in threefold and fourfold increased titers, respectively (Han et al., 2018). The key enzymes in resveratrol synthesis pathway including 4CL1 and STS were recruited to the protein scaffold to relocated in *S. cerevisiae*, and the yield of resveratrol was increased by five times (Wang and Yu, 2012). Through construction and optimization of RNA scaffolds, the two key enzymes in the pentadecane synthetic pathway were spatially colocated to form substrate channel, and the yield of pentadecane was increased by 140% (Gairik et al., 2014). The DNA scaffold can also be used in the production of plant natural products. For example, Conrado constructed a stable and configurable scaffold based on plasmid DNA. Two enzymes 4-coumarate-CoA ligase and STS involved in the resveratrol biosynthesis were arranged and coexpressed via the Zif268 and PBSII ZF domains in the constructed DNA scaffold. As a result, the titer of resveratrol was increased by fivefold. This scaffold can also be used in improving 1,2-propanediol and mevalonate acid production (Conrado et al., 2012). In addition to nucleic acid scaffolds, protein scaffold is also a useful tool to reduce spatial distance by forming substrate channel of multienzyme reactions. The SH3 and PDZ are common ligands in constructing protein scaffold, which are often used in biosynthetic pathway of plant natural products to optimize the intracellular metabolic mass transfer process (Horn and Heinrich, 2015).

### Construction of Metabolic Compartmentalization Enriches the Concentration of Precursors

Heterologous synthesis of plant natural products often brings enormous stress to host cells, such as the accumulation of cytotoxic intermediates and the competition of precursors coupled with the low mass transfer efficiency (Qiu et al., 2019). At the same time, the synthesis of plant natural products usually involves multiple enzymes, and the catalyzed reactions may occur in different locations within the cell, which are affected by the mass transfer efficiency. To avoid these problems and refine the mass transfer, many efficient strategies have been adopted. Among them, spatialized metabolic engineering, especially the metabolic compartmentalization engineering, has been considered as an efficient way. By this method, the whole pathway could be compartmentalized into several modules to enrich the concentration of precursors, and thus the production of plant natural products can be improved. Many organelles are used as metabolon in yeast cell factory, such as mitochondria, peroxisomes, endoplasmic reticulum, and vacuole, which can enhance the mass transfer and provide enough precursors and suitable environment for the synthesis of plant natural products. For example, the sesquiterpene synthetase was relocated in mitochondrial so that the precursors could be catalyzed directly in the same metabolon. Therefore, the production of sesquiterpenoids was increased significantly (Farhi et al., 2011). In a study on squalene production, peroxisomes were harnessed as subcellular compartments to produce the target product, leading to the production of squalene reaching 1,312.8 mg/L, which increased by 138-fold compared with the control strain (Liu et al., 2020). Amorpha-4,11-diene is one of the most important precursors in the artemisinin biosynthesis, starting from acetyl CoA. By colocalizing the FPP biosynthetic pathway (including eight genes) and the amorpha-4,11-diene synthase into mitochondria, the yield of amorpha-4,11-diene was increased significantly as more precursors such as acetyl CoA were provided and mass transfer was enhanced (Yuan and Ching, 2016). To produce isoprene efficiently, its biosynthetic pathway was assembled into mitochondria of yeast cells, and a dual regulation system based on GAL promoter was constructed to control the synthesis. The final isoprene production was 2,527 mg/L in fed-batch fermentation (Lv et al., 2016). Peroxisome is another organelle successfully used in the production of plant natural products. After introducing the farnesyl diphosphate synthetic pathway and α-humulene synthase into the peroxisome, the production of α-humulene was increased by 2.5-fold with a titer of 1,726.78 mg/L in fed-batch fermentation (Zhang et al., 2020). By adopting the modular pathway rewiring strategy, which involved relocalization of the engineered pathway and improving the precursor supply, the titer of L-ornithine reached 1,041 mg/L (Qin et al., 2015).

## Trans-Plasma Membrane Mass Transfer in Yeast Cell Factory

In addition to the intracellular mass transfer, trans-plasma membrane mass transfer also plays a key role in cell metabolism. The intake of nutrients and export of toxic metabolic wastes, as well as secondary metabolites, are the most common transport processes across cell membrane. Moreover, trans-plasma membrane mass transfer is also necessary to synthesize plant natural products. Therefore, enhancing the process of transport is more beneficial for relieving feedback inhibition and toxicity of some products (Agapakis et al., 2012).

### Enhancing Trans-Plasma Membrane Mass Transfer by Transporters

Enhancing the intake of nutrients can provide enough cofactors and precursors to the target pathways. Many uptake transporters have been used to the heterosynthesis of plant natural products. For example, the ATP-binding cassette (ABC) transporter of maltose can enhance the trans-plasma membrane of glucose and maltose. When it was expressed in engineered yeast, the production of ivermectin was improved by obtaining more precursors (Li et al., 2010). Transporter engineering and secretion strengthening of products are among the most effective strategies for improving the production of plant natural products, which not only release the feedback inhibition but also reduce the cytotoxicity. With the development of genome sequencing and metabonomic, the enzymes with new functions (Zhu et al., 2018) together with specific transporters can be mined for the plant natural products. For example, artemisinic acid is the most famous drug to treat malaria, which has been synthesized in *S. cerevisiae* successfully. But in the early development stage, the titer was very low, and many strategies were tried. The pleiotropic drug resistance proteins, which belong to the ABC transporter family, were induced by artemisinic acid. According to the results, it could be speculated that the use of ABC transporters may improve the production of natural products in the engineered yeast (Ro et al., 2008). In addition, the titer of avermectin was also increased two times by overexpression of the ABC transporter AvtAB to enhance the secretory capacity of products (Qiu et al., 2011). However, the research of transporters for plant natural product is still lacking, and the study on secretion of plant natural products in yeast cell factory remains to be further explored.

### Enhancement of Non-specific Trans-Plasma Membrane Mass Transfer

Because of the lack of studies on transporters of plant natural products, some non-specific transport methods have been proposed, including the usage of vesicular, endocytosis, and exocytosis. Vesicle could be used to store tetraterpenes and involves their trans-plasma membrane transport in prokaryote. Regarding the biosynthesis of plant natural products in yeast, vesicle has been proven to be useful as a functional compartment and storage pool. For example, to identify the functional enzyme and improve the titer of tropane alkaloids, more than 20 proteins were integrated into yeast. Among them, the acyltransferase was located to the vacuole to improve the production of products in yeast (Srinivasan and Smolke, 2020). These researches make it possible to enhance trans-plasma membrane transport of plant natural products via vesicles from yeast. Furthermore, the high production of some plant natural products with long synthetic pathway is challenging in a single strain, because of the transmembrane of some intermediate metabolites and the heavy metabolic burden to the yeast cell (Zhang and Wang, 2016; Wang et al., 2020). To reduce the metabolic burden and provide stable environment, the biosynthetic pathway can be divided into several stages and segregated into different strains. Depending on the mass transfer between different cells, it is suitable to produce plant natural products especially terpenoids by constructing *Escherichia coli*–*S. cerevisiae* coculture system. The precursors can be synthesized in *E. coli* and secreted to the medium efficiently. Meanwhile, the secreted precursors are ingested by *S. cerevisiae*, which can provide membrane system and suitable environment for expressing cytochrome P450s (Zhou et al., 2015). For example, paclitaxel is a blockbuster anticancer drug with long and complex biosynthetic pathway. Its *de novo* synthesis in yeast is challenging as some steps of the pathway are not elucidated. Oxygenated taxane is an important intermediate of paclitaxel, whose heterologous biosynthesis has been achieved. In previous study, the *E. coli*–*S. cerevisiae* coculture system was used to increase the titer of oxygenated taxane. In this system, the multistep pathway was divided into several modules to reduce the metabolic burden and facilitate metabolic mass transfer. The engineered *E. coli* provided the important precursor taxadiene, which was then consumed by yeast with inherent membrane system expressing the heterologous P450 monooxygenases (taxadiene 5a-hydroxylase) to produce the oxygenated taxane (Zhou et al., 2015). The opiate is a famous alkaloid compound, whose biosynthetic pathway can be divided into four modules. Galanie constructed a coculture system using four engineered yeasts. The precursors can be synthesized in the first yeast host and transferred into the medium, which could be provided to the next host. Hence, mass transfer optimization by avoiding the degradation of intermediates was achieved, and the production of opiates increased by 300 folds (Galanie et al., 2015). Compared with single strain culturing, coculture of two engineered yeasts achieved higher production of polyketide drug monacolin J and lovastatin with the titer increased by 70% (Liu et al., 2018). Basically, the synthetic pathway of natural products could be divided into several modules. Different modules could be designed and functionalized in different hosts, and the final products can be obtained in the coculture system, which not only reduces the metabolic inhibition of some intermediates but also benefits the yield increase.

## Conclusion and Perspective

Over the past 20 years, the rapid development of synthetic biology and system biology has provided a versatile technical platform for heterologous synthesis of plant natural products. Yeast is turned out to be an attractive host strain. Although various feasible metabolic engineering tools and useful strategies have been developed in yeast to improve the production of plant natural products, there are still many obstacles in the large-scale production. One notable obstacle is the unbalance of metabolic mass transfer process. In this regard, the regulation of metabolic mass transfer has attracted many interests in metabolic engineering applications. For example, regulation and optimization of intracellular and trans-plasma membrane mass transfer can enhance the transfer efficiency, eliminate the feedback inhibition, and reduce the cytotoxicity of products. However, most studies on the optimization of mass transfer process were under specific conditions and lacked of systematization and universality. More importantly, there are seldom successful studies on the mining of direct transporters for plant natural products, which is the main bottleneck for the export of plant natural products in yeast. Thanks to the repaid progress in synthetic biotechnology and sequencing technology, the candidate transporters of plant natural products can be mined by the omics data, bioinformatics analysis and machine learning. Another limitation in the exploring of mass transfer strategies is the lack of high-throughput screening method. As a result, the mining of transporter as well as the integrated platform for high-throughput screening would be future research directions.

In summary, the study of mass transfer has achieved initial success. With the comprehensive progress of the metabolic engineering as well as the genome editing method, the challenges in this field for the synthesis of plant natural products will be solved in the near future. Meanwhile, the mechanisms of metabolic mass transfer process will be revealed and may have a far-reaching significance for constructing a comprehensive and efficient yeast cell factory.

## Author Contributions

All authors contributed to the conception and design of the study. HX and WS participated in searching and analyzing literature for this review and wrote the manuscript. YW and CL edited and corrected the manuscript. All authors approved the submitted version.

## Conflict of Interest

The authors declare that the research was conducted in the absence of any commercial or financial relationships that could be construed as a potential conflict of interest.

## References

[B1] Agapakis C. M. Boyle P. M. Silver P. A. (2012). Natural strategies for the spatial optimization of metabolism in synthetic biology. *Nat. Chem. Biol.* 8 527–535. 10.1038/nchembio.975 22596204

[B2] Albertsen L. Chen Y. Bach L. S. Rattleff S. Maury J. Brix S. (2011). Diversion of flux toward sesquiterpene production in *Saccharomyces cerevisiae* by fusion of host and heterologous enzymes. *Appl. Environ. Microbiol.* 77 1033–1040. 10.1128/AEM.01361-10 21148687 PMC3028695

[B3] Avalos J. L. Fink G. R. Stephanopoulos G. (2013). Compartmentalization of metabolic pathways in yeast mitochondria improves the production of branched-chain alcohols. *Nat. Biotechnol.* 31 335–341. 10.1038/nbt.2509 23417095 PMC3659820

[B4] Conrado R. J. Wu G. C. Boock J. T. Hansen X. Chen S. Y. Tina L. (2012). DNA-guided assembly of biosynthetic pathways promotes improved catalytic efficiency. *Nucleic Acids Res.* 40 1879–1889. 10.1093/nar/gkr888 22021385 PMC3287197

[B5] Enserink M. (2005). Source of new hope against malaria is in short supply. *Science* 307:33. 10.1126/science.307.5706.33 15637249

[B6] Farhi M. Marhevka E. Masci T. Marcos E. Eyal Y. Ovadis M. (2011). Harnessing yeast subcellular compartments for the production of plant terpenoids. *Metab. Eng.* 13 474–481. 10.1016/j.ymben.2011.05.001 21601648

[B7] Gairik S. Abhishek G. David G. Way J. C. Silver P. A. (2014). In vivo co-localization of enzymes on RNA scaffolds increases metabolic production in a geometrically dependent manner. *Nucleic Acids Res.* 42 9493–9503. 10.1093/nar/gku617 25034694 PMC4132732

[B8] Galanie S. Thodey K. Trenchard I. Interrante M. F. Smolke C. D. (2015). Complete biosynthesis of opioids in yeast. *Science* 349 1095–1100. 10.1126/science.aac9373 26272907 PMC4924617

[B9] Han J. Y. Song J. M. Seo S. H. Wang C. Lee S. Lee H. (2018). Ty1-fused protein-body formation for spatial organization of metabolic pathways in *Saccharomyces cerevisiae*. *Biotechnol. Bioeng.* 115 694–704. 10.1002/bit.26493 29131321

[B10] Horn A. H. C. Heinrich S. (2015). Synthetic protein scaffolds based on peptide motifs and cognate adaptor domains for improving metabolic productivity. *Front. Bioeng. Biotechnol.* 3:191. 10.3389/fbioe.2015.00191 26636078 PMC4655305

[B11] Hu T. Zhou J. Tong Y. Su P. Li X. Liu Y. (2020). Engineering chimeric diterpene synthases and isoprenoid biosynthetic pathways enables high-level production of miltiradiene in yeast. *Metab. Eng.* 60 87–96. 10.1016/j.ymben.2020.03.011 32268192

[B12] Khan N. E. Nybo S. E. Chappell J. Curtis W. R. (2015). Triterpene hydrocarbon production engineered into a metabolically versatile host—*Rhodobacter capsulatus*. *Biotechnol. Bioeng.* 112 1523–1532. 10.1002/bit.25573 25728701

[B13] King J. R. Edgar S. Qiao K. Stephanopoulos G. (2016). Accessing nature’s diversity through metabolic engineering and synthetic biology. *F1000Res.* 5:397. 10.12688/f1000research.7311.1 27081481 PMC4813638

[B14] Kirby J. P. Nishimoto M. Chow R. W. N. Pasumarthi V. N. Chan R. Chan L. J. G. (2014). Use of nonionic surfactants for improvement of terpene production in *Saccharomyces cerevisiae*. *Appl. Environ. Microbiol.* 80 6685–6693. 10.1128/AEM.02155-14 25149518 PMC4249056

[B15] Li M. Chen Z. Zhang X. Song Y. Wen Y. Li J. (2010). Enhancement of avermectin and ivermectin production by overexpression of the maltose ATP-binding cassette transporter in *Streptomyces avermitilis*. *Bioresour. Technol.* 101 9228–9235. 10.1016/j.biortech.2010.06.132 20655739

[B16] Li Y. Li S. Thodey K. Trenchard I. Cravens A. Smolke C. D. (2018). Complete biosynthesis of noscapine and halogenated alkaloids in yeast. *Proc. Natl. Acad. Sci. U.S.A.* 115:201721469. 10.1073/pnas.1721469115 29610307 PMC5924921

[B17] Liu G. Li T. Zhou W. Jiang M. Tao X. Liu M. (2020). The yeast peroxisome: a dynamic storage depot and subcellular factory for squalene overproduction. *Metab. Eng.* 57 151–161. 10.1016/j.ymben.2019.11.001 31711816

[B18] Liu Y. Tu X. Xu Q. Bai C. Kong C. Liu Q. (2018). Engineered monoculture and co-culture of methylotrophic yeast for de novo production of monacolin J and lovastatin from methanol. *Metab. Eng.* 45 189–199. 10.1016/j.ymben.2017.12.009 29258964

[B19] Lodish H. F. (1988). Transport of secretory and membrane glycoprotein from the RER to the Golgi. *J. Biol. Chem.* 263 2107–2110. 10.1016/S0021-9258(18)69175-6 3276683

[B20] Lv X. Wang F. Zhou P. Ye L. Xie W. Xu H. (2016). Dual regulation of cytoplasmic and mitochondrial acetyl-CoA utilization for improved isoprene production in *Saccharomyces cerevisiae*. *Nat. Commun.* 7:12851. 10.1038/ncomms12851 27650330 PMC5036000

[B21] Lv Y. Edwards H. Zhou J. Xu P. (2019). Combining 26s rDNA and the Cre-loxP system for iterative gene integration and efficient marker curation in *Yarrowia lipolytica*. *ACS Synth. Biol.* 8 568–576. 10.1021/acssynbio.8b00535 30695641

[B22] Meadows A. L. Hawkins K. M. Tsegaye Y. Antipov E. Kim Y. Raetz L. (2016). Rewriting yeast central carbon metabolism for industrial isoprenoid production. *Nature* 537 694–697. 10.1038/nature19769 27654918

[B23] Mizutani K. Kuramoto T. Tamura Y. Ohtake N. Shigeki D. Nakura M. (1994). Sweetness of glycyrrhetic acid 3-O-β-d-Monoglucuronide and the related glycosides. *Biosci. Biotechnol. Biochem.* 58 554–555. 10.1271/bbb.58.554 7764694

[B24] Moses T. Pollier J. Almagro L. Buyst D. Van Montagu M. Pedreno M. A. (2014). Combinatorial biosynthesis of sapogenins and saponins in *Saccharomyces cerevisiae* using a C-16α hydroxylase from Bupleurum falcatum. *Proc. Natl. Acad. Sci. U.S.A.* 111 1634–1639. 10.1073/pnas.1323369111 24434554 PMC3910630

[B25] Noorman H. J. (2001). “Mass transfer,” *Basic Biotechnology*, ed. Ratledge C. (New York, NY: Cambridge university press), 201–218.

[B26] Nour A. M. M. Khalid S. A. Kaiser M. Brun R. Abdallah W. E. Schmidt T. J. (2009). The antiprotozoal activity of sixteen asteraceae species native to sudan and bioactivity-guided isolation of Xanthanolides from *Xanthium brasilicum*. *Planta Med.* 75 1363–1368. 10.1055/s-0029-1185676 19431098

[B27] Paddon C. J. Westfall P. J. Pitera D. J. Benjamin K. R. Fisher K. Mcphee D. J. (2013). High-level semi-synthetic production of the potent antimalarial artemisinin. *Nature* 496 528–532. 10.1038/nature12051 23575629

[B28] Park S. R. Yoon J. A. Paik J. Park J. W. Jung W. S. Ban Y. (2009). Engineering of plant-specific phenylpropanoids biosynthesis in *Streptomyces venezuelae*. *J. Biotechnol.* 141 181–188. 10.1016/j.jbiotec.2009.03.013 19433224

[B29] Peraltayahya P. Ouellet M. Chan R. Mukhopadhyay A. Keasling J. D. Lee T. S. (2011). Identification and microbial production of a terpene-based advanced biofuel. *Nat. Commun.* 2:483. 10.1038/ncomms1494 21952217 PMC3195254

[B30] Poshyvailo L. Lieres E. V. Kondrat S. (2017). Does metabolite channeling accelerate enzyme-catalyzed cascade reactions? *PLos One* 12:e0172673. 10.1371/journal.pone.0172673 28234973 PMC5325314

[B31] Qin J. Zhou Y. J. Krivoruchko A. Huang M. Liu L. Khoomrung S. (2015). Modular pathway rewiring of *Saccharomyces cerevisiae* enables high-level production of L-ornithine. *Nat. Commun.* 6:8224. 10.1038/ncomms9224 26345617 PMC4569842

[B32] Qiu C. Zhai H. Hou J. (2019). Biosensors design in yeast and applications in metabolic engineering. *FEMS Yeast Res.* 8:foz082. 10.1093/femsyr/foz082 31778177

[B33] Qiu J. Zhuo Y. Zhu D. Zhou X. Zhang L. Bai L. (2011). Overexpression of the ABC transporter AvtAB increases avermectin production in *Streptomyces avermitilis*. *Appl. Microbiol. Biotechnol.* 92 337–345. 10.1007/s00253-011-3439-4 21713508

[B34] Reddingjohanson A. M. Batth T. S. Chan R. Krupa R. A. Szmidt H. L. Adams P. D. (2011). Targeted proteomics for metabolic pathway optimization: application to terpene production. *Metab. Eng.* 13 194–203. 10.1016/j.ymben.2010.12.005 21215324

[B35] Ro D. Ouellet M. Paradise E. M. Burd H. Eng D. Paddon C. J. (2008). Induction of multiple pleiotropic drug resistance genes in yeast engineered to produce an increased level of anti-malarial drug precursor, artemisinic acid. *BMC Biotechnol.* 8:83. 10.1186/1472-6750-8-83 18983675 PMC2588579

[B36] Ro D. K. Douglas C. J. (2004). Reconstitution of the entry point of plant phenylpropanoid metabolism in yeast (*Saccharomyces cerevisiae*): implications for control of metabolic flux into the phenylpropanoid pathway. *J. Biol. Chem.* 279 2600–2607. 10.1074/jbc.M309951200 14607837

[B37] Shan H. Segura M. J. R. Wilson W. K. Lodeiro S. Matsuda S. P. T. (2005). Enzymatic cyclization of dioxidosqualene to heterocyclic triterpenes. *J. Am. Chem. Soc.* 127 18008–18009. 10.1021/ja055822g 16366544

[B38] Smith J. J. Brown T. W. Eitzen G. A. Rachubinski R. A. (2000). Regulation of peroxisome size and number by fatty Acid β-Oxidation in the yeast *Yarrowia lipolytica*. *J. Biol. Chem.* 275 20168–20178. 10.1074/jbc.M909285199 10787422

[B39] Srinivasan P. Smolke C. D. (2020). Biosynthesis of medicinal tropane alkaloids in yeast. *Nature* 585 614–619. 10.1038/s41586-020-2650-9 32879484 PMC7529995

[B40] Su S. Liu X. Pan G. Hou X. Zhang H. Yuan Y. (2015). In vitro characterization of a (E)-β-farnesene synthase from *Matricaria recutita* L. and its up-regulation by methyl jasmonate. *Gene* 571 58–64. 10.1016/j.gene.2015.06.037 26095800

[B41] Sun W. Xue H. Liu H. Lv B. Yu Y. Wang Y. (2020). Controlling chemo- and regioselectivity of a plant P450 in yeast cell toward rare licorice triterpenoid biosynthesis. *ACS Catal.* 10 4253–4260. 10.1021/acscatal.0c00128

[B42] Trantas E. A. Panopoulos N. J. Ververidis F. (2009). Metabolic engineering of the complete pathway leading to heterologous biosynthesis of various flavonoids and stilbenoids in *Saccharomyces cerevisiae*. *Metab. Eng.* 11 355–366. 10.1016/j.ymben.2009.07.004 19631278

[B43] Valachovic M. Garaiova M. Holic R. Hapala I. (2016). Squalene is lipotoxic to yeast cells defective in lipid droplet biogenesis. *Biochem. Biophys. Res. Commun.* 469 1123–1128. 10.1016/j.bbrc.2015.12.050 26703208

[B44] Wang R. Zhao S. Wang Z. Koffas M. A. G. (2020). Recent advances in modular co-culture engineering for synthesis of natural products. *Curr. Opin. Biotechnol.* 62 65–71. 10.1016/j.copbio.2019.09.004 31605875

[B45] Wang Y. Yu O. (2012). Synthetic scaffolds increased resveratrol biosynthesis in engineered yeast cells. *J. Biotechnol.* 157 258–260. 10.1016/j.jbiotec.2011.11.003 22100267

[B46] Wheeldon I. Minteer S. D. Banta S. Barton S. C. Atanassov P. Sigman M. (2016). Substrate channelling as an approach to cascade reactions. *Nat. Chem.* 8 299–309. 10.1038/nchem.2459 27001725

[B47] Whittam R. Wheeler K. P. (1970). Transport across cell membranes. *Annu. Rev. Physiol.* 32 21–60. 10.1146/annurev.ph.32.030170.000321 4244435

[B48] Williams D. H. Stone M. J. Hauck P. R. Rahman S. K. (1989). Why are secondary metabolites (natural products) biosynthesized. *J. Nat. Prod.* 52 1189–1208. 10.1021/np50066a001 2693613

[B49] Xu Y. Liu Y. Rasool A. Wenwen E. Li C. (2017). Sequence editing strategy for improving performance of β-glucuronidase from *Aspergillus terreus*. *Chem. Eng. Sci.* 167 145–153. 10.1016/j.ces.2017.04.011

[B50] Yang X. Nambou K. Wei L. Hua Q. (2016). Heterologous production of α-farnesene in metabolically engineered strains of *Yarrowia lipolytica*. *Bioresour. Technol.* 216 1040–1048. 10.1016/j.biortech.2016.06.028 27347651

[B51] Yuan J. Ching C. (2016). Mitochondrial acetyl-CoA utilization pathway for terpenoid productions. *Metab. Eng.* 38 303–309. 10.1016/j.ymben.2016.07.008 27471067

[B52] Zhang C. Li M. Zhao G. R. Lu W. (2020). Harnessing yeast peroxisomes and cytosol Acetyl-CoA for sesquiterpene alpha-humulene production. *J. Agric. Food Chem.* 68 1382–1389. 10.1021/acs.jafc.9b07290 31944688

[B53] Zhang H. Wang X. (2016). Modular co-culture engineering, a new approach for metabolic engineering. *Metab. Eng.* 37 114–121. 10.1016/j.ymben.2016.05.007 27242132

[B54] Zhang Y. Li S. Z. Li J. Pan X. Cahoon R. E. Jaworski J. G. (2006). Using unnatural protein fusions to engineer resveratrol biosynthesis in yeast and mammalian cells. *J. Am. Chem. Soc.* 128 13030–13031. 10.1021/ja0622094 17017764

[B55] Zhao J. Bao X. Li C. Shen Y. Hou J. (2016). Improving monoterpene geraniol production through geranyl diphosphate synthesis regulation in *Saccharomyces cerevisiae*. *Appl. Microbiol. Biotechnol.* 100 4561–4571. 10.1007/s00253-016-7375-1 26883346

[B56] Zhou K. Qiao K. Edgar S. Stephanopoulos G. (2015). Distributing a metabolic pathway among a microbial consortium enhances production of natural products. *Nat. Biotechnol.* 33 377–383. 10.1038/nbt.3095 25558867 PMC4867547

[B57] Zhou Y. J. Gao W. Rong Q. Jin G. Chu H. Liu W. (2012). Modular pathway engineering of diterpenoid synthases and the mevalonic acid pathway for miltiradiene production. *J. Am. Chem. Soc.* 134 3234–3241. 10.1021/ja2114486 22280121

[B58] Zhu M. Wang C. Sun W. Zhou A. Wang Y. Zhang G. (2018). Boosting 11-oxo-beta-amyrin and glycyrrhetinic acid synthesis in *Saccharomyces cerevisiae* via pairing novel oxidation and reduction system from legume plants. *Metab. Eng.* 45 43–50. 10.1016/j.ymben.2017.11.009 29196123

